# Distribution and diversity of mycoplasma plasmids: lessons from cryptic genetic elements

**DOI:** 10.1186/1471-2180-12-257

**Published:** 2012-11-12

**Authors:** Marc Breton, Florence Tardy, Emilie Dordet-Frisoni, Eveline Sagne, Virginie Mick, Joël Renaudin, Pascal Sirand-Pugnet, Christine Citti, Alain Blanchard

**Affiliations:** 1 University Bordeaux, UMR 1332 Biologie du Fruit et Pathologie, 71 avenue Edouard Bourlaux, F-33140, Villenave d'Ornon, France; 2 INRA, UMR 1332 Biologie du Fruit et Pathologie, 71, avenue Edouard Bourlaux, F-33140, Villenave d'Ornon, France; 3 Anses, Laboratoire de Lyon, UMR Mycoplasmoses des Ruminants, 31 Avenue Tony Garnier, F-69364, Lyon cedex 07, France; 4 INRA, UMR1225, Ecole Nationale Vétérinaire de Toulouse, 23 Chemin des Capelles, F-31076, Toulouse Cedex 3, France; 5 Université de Toulouse, INP-ENVT, UMR1225, Ecole Nationale Vétérinaire de Toulouse, 23 Chemin des Capelles, F-31076, Toulouse Cedex 3, France; 6 Centre INRA de Bordeaux Aquitaine, UMR 1332 Biologie du Fruit et Pathologie, 71, avenue Edouard Bourlaux, BP81, F-33140, Villenave d'Ornon, France

**Keywords:** Mycoplasma,Plasmid,Replication,Rep protein,Gene transfer,Evolution,Expression vector,*Mycoplasma mycoides*,*Mycoplasma capricolum*,*Mycoplasma yeatsii*

## Abstract

**Background:**

The evolution of mycoplasmas from a common ancestor with *Firmicutes* has been characterized not only by genome down-sizing but also by horizontal gene transfer between mycoplasma species sharing a common host. The mechanisms of these gene transfers remain unclear because our knowledge of the mycoplasma mobile genetic elements is limited. In particular, only a few plasmids have been described within the *Mycoplasma* genus.

**Results:**

We have shown that several species of ruminant mycoplasmas carry plasmids that are members of a large family of elements and replicate via a rolling-circle mechanism. All plasmids were isolated from species that either belonged or were closely related to the *Mycoplasma mycoides* cluster; none was from the *Mycoplasma bovis-Mycoplasma agalactiae* group. Twenty one plasmids were completely sequenced, named and compared with each other and with the five mycoplasma plasmids previously reported. All plasmids share similar size and genetic organization, and present a mosaic structure. A peculiar case is that of the plasmid pMyBK1 from *M. yeatsii;* it is larger in size and is predicted to be mobilizable. Its origin of replication and replication protein were identified. In addition, pMyBK1 derivatives were shown to replicate in various species of the *M*. *mycoides* cluster, and therefore hold considerable promise for developing gene vectors. The phylogenetic analysis of these plasmids confirms the uniqueness of pMyBK1 and indicates that the other mycoplasma plasmids cluster together, apart from the related replicons found in phytoplasmas and in species of the clade *Firmicutes.*

**Conclusions:**

Our results unraveled a totally new picture of mycoplasma plasmids. Although they probably play a limited role in the gene exchanges that participate in mycoplasma evolution, they are abundant in some species. Evidence for the occurrence of frequent genetic recombination strongly suggests they are transmitted between species sharing a common host or niche.

## Background

Horizontal gene transfer (HGT) is recognized as the major force in bacterial genome evolution (for review see: [1]). It has contributed to the diversity of bacterial species and to the success of bacterial colonization of almost all the possible niches on earth. HGT events have been detected in most bacteria for which genome sequences are available. Yet many questions remain about the dynamics of gene exchange and the mechanisms underlying these DNA transfers. Some bacterial species seem particularly well equipped for sharing DNA at high frequency (for review see: [2]). These bacteria present an abundance of different mobile genetic elements (MGE) and have other characteristics such as natural competence, efficient machinery for homologous recombination and numerous secretion systems that favor gene exchange. For other bacteria with limited MGE repertoire and routes of DNA transfer, the means of genetic exchange are not so obvious.

The class *Mollicutes* is a group of wall-less bacteria that colonize a variety of hosts, from plants to humans, and are characterized by a small genome with a low G+C content [3,4]. *Mollicutes* are thought to have evolved from a common ancestor with *Firmicutes* through successive genome losses [5]. This drastic evolution resulted in some mollicutes, such as *Mycoplasma genitalium,* having a cell with a highly reduced genome that is considered the best representative of a natural minimal cell [6]. However, genome down-sizing was not the sole force operating during evolution because it has been shown that mollicutes were also able to exchange genetic material through HGT. Indeed, comparative genomics of ruminant mycoplasmas predicted that up to 18% of the *Mycoplasma agalactiae* genome has undergone HGT with mycoplasmas of the distinct *Mycoplasma mycoides* cluster [4]. A smaller amount of HGT has also been detected between two bird pathogens *M. gallisepticum* and *M. synoviae,* and between two human urogenital pathogens, *M. hominis* and *Ureaplasma parvum*[7,8]. Obviously, sharing a common host was a requisite for HGT but the underlying mechanisms behind these HGT events have yet to be described. A number of MGE, including integrative and conjugative elements (ICEs), insertion sequences (IS), phages and plasmids, have been described in these bacteria and are potential candidates for mediating these genetic transfers.

**Figure 1:**
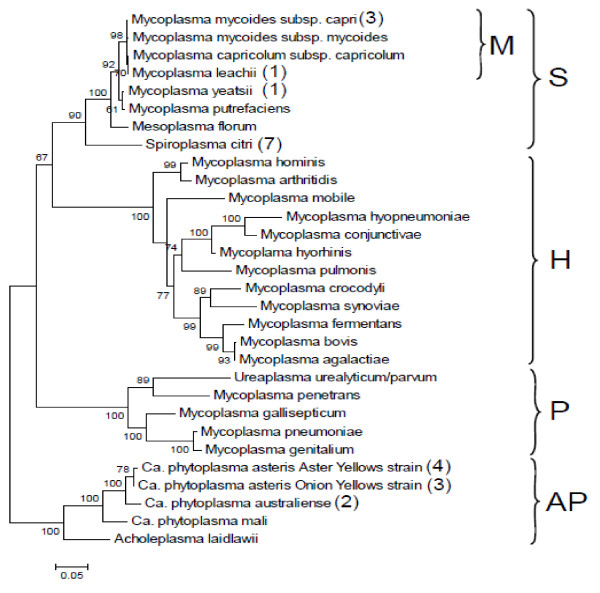
**Mollicute phylogenetic tree including species for which at least one genome sequence is available.** The mollicute evolutionary history was inferred by using the Maximum Likelihood method based on the Tamura-Nei model [9]. The tree with the highest log likelihood (−8994.2924) is shown. The percentage of trees in which the associated taxa clustered together is shown next to the branches. Initial tree(s) for the heuristic search were obtained automatically as follows. When the number of common sites was < 100 or less than one fourth of the total number of sites, the maximum parsimony method was used; otherwise BIONJ method with MCL distance matrix was used. A discrete Gamma distribution was used to model evolutionary rate differences among sites (5 categories (+*G*, parameter = 0.5355)). The tree is drawn to scale, with branch lengths measured in the number of substitutions per site. Nucleotide sequences (16S rDNA) from 30 species were aligned. After removing all positions containing gaps and missing data, the final dataset included 1136 positions.Evolutionary analyses were conducted in MEGA5 [10]. The number in parentheses indicates the number of plasmids previously described for each species. No indication means that there is no reported evidence of plasmid in these species. For *M. mycoides* subsp. *capri*, each one of the three plasmids was identified in a different strain. The letters on the right side of the figure indicate the phylogenetic groups within the *Mollicutes*: S, Spiroplasma; H: Hominis; P: Pneumoniae; AP: Acholeplasma-Phytoplasma; M: *Mycoplasma mycoides* cluster.

Although usually abundant in species belonging to the phylum *Firmicutes*, only a few plasmids have been described in the different genera of the *Mollicutes* (Figure 1). They were first detected in the genus *Spiroplasma*[11,12] and later proved widely distributed in this genus [13]. Spiroplasma plasmids that have a size ranging from 5 to more than 30 kbp were initially termed cryptic as no specific phenotype was associated with their presence. However, some of these plasmids carry genetic determinants that play a role in the transmission of the *Spiroplasma citri* by its vector insect [14,15]. Within *Mollicutes,* the other phytopathogen organisms are phytoplasmas that remain yet uncultivated. In several *Candidatus* phytoplasma species, plasmids with a size range from 2.6 to 10.8 kbp have also been described (for a review see [16]). Unlike the spiroplasma plasmids for which no homology was detected in databases, all the phytoplasma plasmids encode a replication protein sharing similarities with the Rep proteins involved in rolling-circle replication (RCR) [17,18]. For the genus *Mycoplasma,* which includes over 100 species, among which are significant pathogens of animals and humans [19], only five plasmid sequences are available in databases [20-23] (Figure 1). All 5 plasmids have been isolated in *Mycoplasma* species belonging to the Spiroplasma phylogenetic group but are not related to the ones described in *Spiroplasma* species. Four are from closely related species of the *M. mycoides* cluster and three of them (pADB201, pKMK1, and p*Mmc*-95010) are from the same sub-species, *M. mycoides* subsp. *capri (Mmc)*. In contrast to the apparent scarcity of mycoplasma plasmids, other investigators have reported a much higher prevalence of strains with plasmids but these data were only based on agarose gel detection of extrachromosomal DNA, without DNA sequencing [24].

The present work was conducted in order to better comprehend the nature and extend of the plasmid repertoire of two main groups of ruminant mycoplasmas: the *M. agalactiae-M. bovis* group and the species found within or close to the *M. mycoides* cluster, two phylogenetically distant groups between which a high level of HGT has been predicted in silico [4] (Figure 1). Several plasmids were isolated from various species and completely sequenced. Comparative analyses indicated that, except for the recently described pMyBK1 from *M. yeatsii*[25], all plasmids belong to the same large family of rolling-circle replicons found in *Firmicutes.* Plasmid pMYBK1 represents a new family of replicons that can be transformed and maintained in other mycoplasma species. The study further indicates that plasmids can be commonly found in several *Mycoplasma* species colonizing ruminants and therefore, could contribute to the genetic transfers that have been revealed by comparative genomics.

## Methods

### Mycoplasma strains, growth conditions and DNA purification

All mycoplasma strains used in this study (Table 1) are kept in the collection maintained by the Anses laboratory of Lyon and most of them were isolated as part of the activities of the Vigimyc network [26]. They were cultivated at 37°C in Mycoplasma broth base supplemented as for SP4 medium [27]. Mycoplasma transformants were sub-cultured in modified Hayflick broth [28] supplemented with 0.4% (w/v) pyruvate, 0.2% (w/v) glucose and 5–15 μg of tetracycline mL^-1^. *Spiroplasma citri* was grown at 32°C in SP4 broth withoutfresh yeast extract. *Escherichia coli* DH10B was used as the host strain in cloning experiments and was grown in LB medium supplemented with 100 μg.ml^-1^ of ampicillin for selection.

**Table 1:** Mycoplasma plasmids analyzed in this study

**Taxon**	**Strain name**	**Plasmid name**	**Reference**	**GenBank access n°**	**Plasmid size**
*M. leachii*	99/0361	pBG7AU	Djordjevik et al. 2001	AF257325	1022 bp
	CIRAD063*^++^	pBG7AU	this work	/	1022 bp
*Mmc*	GM12	pKMK1	King & Dybvig, 1992	M81470	1875bp
	GC1176-2	pADB201	Bergemann et al. 1989	M25059	1717 bp
	95010	p*Mmc*-95010	Thiaucourt et al. 2011	FQ790215	1840 bp
	13071	p*Mmc*-95010-3	this work	/^a^	1839 bp
	14227	pMG1A-1	this work	JX294729	1865 bp
	L	p*Mmc*-95010-2	this work	/	1802 bp
	4343	pMG1C-1	this work	JX294730	1770 bp
*M. yeatsii*	GIH (TS)	pMyBK1	Kent et al. 2012	EU429323	3432 bp
	GIH (TS)	pMG2B-1	this work	JX294731	1573bp
	11181	pMG2F-1	this work	JX294732	1656 bp
	15000	pMG2F-2	this work	/	1652 bp
*M. cottewii*	VIS (TS)	pMG2C-1	this work	JX294733	1565 bp
	15104	pMG2E-1	this work	JX294734	1041 bp
*Mcc*	14425	pMG1B-1	this work	JX294737	1732bp
	14667	pMG1B-2	this work	/	1731 bp
	15301	pMG1B-3	this work	/	1731 bp
	5145	pMG1B-4	this work	/	1733 bp
	15250	pMG1B-5	this work	/	1732 bp
	15216	pMG1B-6	this work	/	1734 bp
	14250	pMG2A-1	this work	JX294735	1573 bp
	11186	pMG2D-1	this work	JX294736	1722 bp
	14141	pMG2D-2	this work	/	1720 bp
	14332	pMG2D-3	this work	/	1718 bp
	4142	pMG2D-4	this work	/	1720 bp

^a^ the sequences for which the plasmid is the representative of a series have been deposited in GenBank.

Mycoplasma and spiroplasma genomic DNA were prepared using the Wizard Genomic DNA Purification kit (Promega) or by standard phenol/chloroform procedures. Plasmid DNA was purified using either the Wizard SV Minipreps DNA purification kit (Promega) or QIAprep Spin Miniprep Kit (Qiagen) with the low-copy plasmid protocol. When several plasmids were present, as in *M. yeatsii* GIH TS, the individual bands visualized on agarose gel were purified following an agarase (AgarACE™, Promega) treatment.

### Screening mycoplasma strains for the presence of plasmids

The presence of plasmid was screened by agarose gel electrophoresis of 1 μg of genomic DNA extracted from cells collected from stationary phase cultures.

### Determination of plasmid copy number

The copy number of pMyBK1 and pMG2B-1 was estimated by gel assay as previously described [29] except that lysozyme treatment was omitted. Serial twofold dilutions of the genomic DNA extracted from a logarithmic phase culture of *M. yeatsii* GIH^T^ were analyzed by 0.8% (w/v) agarose gel electrophoresis. After ethidium bromide staining, the relative intensities of individual bands, both plasmid and chromosome, were quantified using the ImageJ software [30]. The copy numbers of pMyBK1 and pMG2B-1 were derived from the intensity of each band taking into account their respective sizes. The plasmid copy number was also quantified by real-time PCR as reported earlier by others [31]. Amplification and detection were carried out using a Roche LightCycler 480 (Roche Diagnostics) using a SYBR green/fluorescein mix (Applied Biosystem). The glycerol kinase gene *glpk* was chosen as the reference gene, because it is a conserved single-copy gene that is chromosomally encoded. Fragments of chromosomal *glpk* (123 bp)*,* pMyBK1 *cdsB* (90 bp) and pMG2B-1 *rep* (87 bp) were amplified with primers glpkF/R, cdsBF/R and pMG2B-1F/R, respectively (Additional file 1: Table S1). The amplification efficiencies were determined through serial tenfold dilutions of the DNA samples using the LightCycler 480 software and were shown to be similar for each target gene, namely *glpk, cdsB* and *rep*. The relative copy number N of pMyBK1 or pMG2B-1 plasmids was calculated by the following formula: N relative = (1+E)-ΔCt, where E and ΔCt represent the PCR amplification efficiency and the difference between the cycle threshold number (Ct) of *glpk* and *cdsB* or *rep* reaction, respectively. The experiment was performed in triplicate.

### DNA sequencing and sequence analyses

Purified mycoplasma plasmids were linearized using a restriction enzyme (*Eco*RI, *Eco*RV or *Hin*dIII) and were then sub-cloned into the pBluescript vector linearized with the same enzyme. The resulting plasmids were sequenced using T7 and T3 universal primers or by primer-walking when necessary. When there was not a unique restriction site within the plasmid, multiple restriction fragments were individually sub-cloned and sequenced. The nucleotide sequences were determined by means of at least two overlapping reads on each strand of the whole plasmids. When necessary, complementary plasmid sequences were obtained by direct sequencing of PCR products (for the list of PCR primers see Additional file 1: Table S1). The plasmid sequences determined in this study have been deposited in the GenBank database under the following accession numbers: JX294729 for pMG1A-1, JX294730 for pMG1C-1, JX294731 for pMG2B-1, JX294732 for pMG2F-1, JX294733 for pMG2C-1, JX294734 for pMG2E-1, JX294735 for pMG2A-1, JX294736 for pMG2D-1 and JX294737 for pMG1B-1 (Table 1).

Coding sequences (CDSs) were searched using the AMIGene software ([32], http://www.genoscope.cns.fr/agc/tools/amigene/). Database searches and comparisons of DNA sequences or DNA-derived protein sequences were carried out using BLAST programs (http://www.ncbi.nlm.nih.gov/blast/). Conserved domains were detected by CD-Search against the CDD resource from NCBI [33]. Protein secondary structures were predicted from sequences using the SOPM method [34]. DNA repeats were identified using the software RepFind [35], nucleic acid folding and calculation of free energy for hairpin formation were determined using the Mfold program [36]. Multiple sequence alignments were performed with T-Coffee [37] or ClustalW2 softwares [38]. Subsequent phylogenetic analyses were performed with the Mega 5 software [10] using the neighbor-joining or the maximum likelihood method. Multiple-way pairwise comparisons of plasmid nucleic sequences were conducted with the Artemis Comparison Tool, ACT [39].

### Southern blot hybridization and immunoblotting

The detection of ssDNA intermediates was performed by Southern blot hybridization and S1 nuclease treatment as described previously by others [40]. Total *M. yeatsii* GIH TS genomic DNA, treated or not with S1 nuclease (10 U/μg of DNA for 15 min at room temperature) was electrophoresed using a 0.8% agarose gel and transferred without prior denaturation to a nylon membrane (Nytran SuPerCharge) by vacuum blotting in 10X SSC buffer (Vacuum Blotter; MP Biomedicals). The air-dried membrane was then UV cross-linked before hybridization with the pMyBK1 [digoxigenin]dUTP-labelled probe using standard stringency conditions. Hybridization signals were detected with anti-digoxigenin-alkaline phosphatase conjugate and CDP-Star as the substrate, according to the manufacturer's instructions (Roche Applied Science). The pMyBK1 probe was generated by PCR amplification with primer pair pMyBK1-F1/R2 (Additional file 1: Table S1).

For protein immunobloting, 10^7^–10^8^ c.f.u. from *M. yeatsii* and *M. capricolum* subsp. *capricolum* (*Mcc*) late-exponential-phase cultures were spotted under vacuum onto a nitrocellulose membrane. Immunoblotting was carried out as described previously [41] except that the binding of spiralin-antibodies was visualized by using a goat anti-rabbit immunoglobulin G–peroxidase conjugate and the Super Signal West Pico chemoluminescent substrate (Pierce).

### Plasmid constructs and transformation experiments

**Figure 2:**
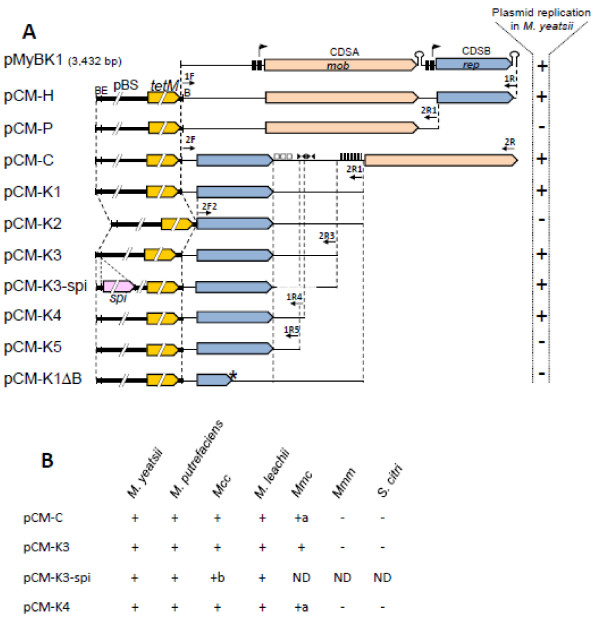
**Structural organization and replication ability of pMyBK1 and derivatives.A.** Plasmid constructs are described in Methods. Putative promoter and terminator of CDSA and CDSB are indicated for pMyBK1 only. Direct repeats (□) , inverted repeats (▸◂) and the GC-rich region (|||||) are indicated only for the pCM-C derivative. B, *Bgl*II; E, *Eco*RI; *spi*, *Spiroplasma citri* spiralin gene; *tetM*, tetracycline resistance gene from transposon Tn*916*, pBS, plasmid pBluescript. The signs on the right indicate the ability (+) and inability (−) to replicate in *Mycoplasma yeatsii* type strain GIH TS. * indicates a frameshift mutation in the *cdsB* sequence of pCM-K1ΔB. **B.** The replication ability of 4 pMyBK1 derivatives was evaluated in mollicute species belonging to the Spiroplasma phylogenetic group and shown to be initially plasmid-free: *M. yeatsii* #13156, *M. putrefaciens* KS1 TS, *M. leachii* PG50 TS, *Mcc* California kid TS, *Mmc* GM12, *Mmm* T1/44 and *S. citri* GII3. The signs on the right indicate the ability (+) and inability (−) to replicate in a given species. ND: not determined. **A**: plasmid integration in the *Mmc* chromosome. **B**: spiralin expression in *Mcc* was detected by immunoblot.

Several derivatives of pMyBK1 (pCM-H, pCM-P, pCM-C, pCM-K1-5) were constructed by inserting *Bgl*II-digested amplification products from pMyBK1 (*Bgl*II site in the primer sequences) into *Bgl*II-linearized pSRT2 [42]. Primers used for amplification of fragments from pMyBK1 are listed in Additional file 1: Table S1. In each construct (see Results section and Figure 2), the CDSs of pMyBK1 were kept in the same orientation as that of the pSRT2 *tetM* gene. To produce pCM-K3-spi, the spiralin gene and its promoter were amplified from *S. citri* GII3 genomic DNA with primer pair SpiERI-F/R, prior to restriction with *Eco*RI and ligation into *Eco*RI-linearized pCM-K3. In pCM-K1ΔB, the CDSB of pCM-K1 was disrupted by a 4-bp insertion creating a unique *Xho*I site. To introduce the 4-bp frameshift mutation, the amplification product of pCM-K1 using DeltacdsB-F/DeltacdsB-R primers was restricted by *Xho*I before circularization by self-ligation.

Electrotransformation of *S. citri* was carried out as previously described [43] with 1–5 μg of DNA. Polyethylene glycol-mediated transformation of mycoplasmas was performed as described previously [44] with 5–10 μg of plasmid and transformants were selected by plating on medium containing 5–15 μg.ml^−1^ of tetracycline.

## Results and discussion

### Detection and initial characterization of plasmids from ruminant mycoplasmas

A total of 194 ruminant mycoplasma strains were selected from our collection on the basis that there was no apparent epidemiological link between them. Their distribution amongst taxa is summarized in Table 2. No plasmid was detected in species belonging to the Hominis phylogenetic group, i.e. in the *M. bovis* and *M. agalactiae* species. In contrast, several plasmids were detected in strains belonging to the *M. mycoides* cluster or to closely related species of the Spiroplasma phylogenetic group (Table 2). Indeed, 37 out of the 112 strains screened (33%) were found to carry plasmids. Although plasmids have already been described for strains belonging to the *Mmc, M. yeatsii* and *M. leachii* species, this is the first report of plasmids in *M. cottewii* and *Mcc*. While nearly all strains carried a single plasmid, the *M. yeatsii* (GIH) type strain contained two plasmids. Except for the larger plasmid of *M. yeatsii* GIH TS (3.4 kbp), all other plasmids had apparent sizes of 1.0 to 2.0 kbp. Also, no correlation between the presence of plasmid and the history of the strains such as the year and/or place of isolation, and the host species (bovine *versus* caprine), could be established (Additional file 2: Table S2).

**Table 2:** Detection of plasmids from ruminant mycoplasmas

**Phylogenetic group**	**Taxon**	**nb of screened strains **^**a**^	**strains with plasmid**^**b**^
Hominis	*M. agalactiae*	40	0
	*M. bovis*	42	0
	Subtotal	82	0
Spiroplasma	*M. mycoides* subsp. *capri*	43	12
	*M. capricolum* subsp. *capricolum*	41	15
	*M. leachii*	10	1
	*M. yeatsii*	16	7
	*M. cottewii*	2	2
	Subtotal	112	37
	Total	194	37

(a) including the species type strain.

(b) as visualized on agarose gel after total DNA extraction by phenol/chloroform.

Twenty one plasmids, at least one per taxon, were randomly chosen and fully sequenced. Plasmid sizes ranged from 1,041 bp to 1,865 bp. To assess the diversity and genetic variability of mycoplasma plasmids, the 21 sequences were compared to each other and to those of the five mycoplasma plasmids available in GenBank: pADB201, pKMK1, and p*Mmc*95010 from *Mmc*, pBG7AU from *M. leachii*, and pMyBK1 from *M. yeatsii* (Table 1)*.* The overall nucleotide identity was calculated after a global alignment for each plasmid-pair. Within individual taxa, pairwise nucleotide identities varied from 100% to less than 40% (Additional file 3: Table S3). Indeed, the sequence of the plasmid that we isolated from a *M. leachii* strain was found to be identical to that of the previously described pBG7AU. This result is not surprising since the 2 *M. leachii* strains, though distinct, were recovered from the same outbreak in Australia [21]. Similarly, the 2 field strains of *M. yeatsii* were shown to harbor plasmids that are 97% identical. In this case, however, the strains sharing the same geographical origin were isolated 8 years apart. In contrast, the 2 plasmids isolated from the *M. cottewii* species were shown to have different sizes (1,565 vs 1,041 bp) and nucleotide sequences (42% identity only). The pMyBK1 plasmid, sequenced by others (Genbank accession # EU429323; [25]) and also found in the *M. yeatsii* type strain, is certainly a particular case because of its larger size (3,422 bp) and low nucleotide identity (20-37%) in comparison to other mycoplasma plasmids.

### Proposed nomenclature for mycoplasma plasmids

With the description of this fairly large set of plasmids, a proposal for a new nomenclature of mycoplasma plasmids seemed justified. First, we considered that there was no need to give a different name to a plasmid that was found identical to a previously described replicon (e.g. pBG7AU). For the plasmids that are very close to each other (nucleotide identity & 95%), we considered that they were variants and should be given the same name followed by the suffix “-n” where n indicated the number by chronological order in this series of plasmids (Table 1); the plasmid with the suffix “-1” being the prototype of the plasmid series (e.g. pMG1A-1). This same rule was used for variants of plasmids described by others (e.g. p*Mmc*-95010-2). Finally, the plasmids were separated into two groups (G1 and G2) according to their *rep* sequences (see below). According to this nomenclature, we identified 9 new plasmids (pMG1A-1, pMG1B-1, pMG1C-1, pMG2A-1, pMG2B-1, pMG2C-1, pMG2D-1, pMG2E-1 and pMG2F-1) and 11 variants of these plasmids or of plasmids previously reported. Sequences of these 9 new plasmids have been deposited in GenBank (Table 1).

### Mycoplasma plasmids share a common genetic organization

**Figure 3:**
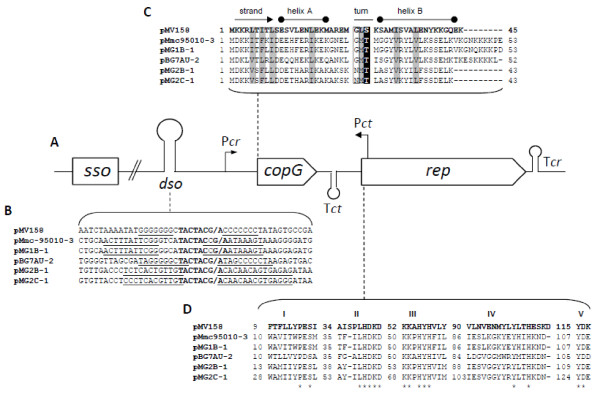
**Molecular features of mycoplasma plasmids of the pMV158 family.A.** Typical genetic organisation of the replication region of plasmids belonging to the pMV158 family. Putative promoter and terminator of *coprep* RNA (Pcr, Tcr), leading strand origin of replication (dso), region predicted to include the lagging-strand initiation site (sso) and ctRNA (Pct, Tct) are indicated. Not drawn to scale. **B.** Comparison of double strand origins. The inverted repeats are underlined. Conserved nucleotides in nick sequences are indicated by bold letters. (/) denote nick site by RepB in pMV158 and the putative nick sites in mycoplasma plasmids. **C.** Multiple sequence alignments of CopG proteins. Conserved hydrophobic positions are shaded and the conserved Thr/Ser is marked with white font on black background. Boxed letters represent the conserved Gly/Asx residue of the turn connecting helix A and B. **D.** Multiple sequence alignments of Rep proteins. Motifs typical of pMV158 plasmid family are shown according to del Solar et al. [46]. Numbers indicate positions of the motifs in the Rep sequences and asterisks indicate the conserved position in all aligned Rep sequences.

With the exception of pMyBK1for which a specific analysis is provided further, all plasmids shared the same overall genetic organization, similar to those of p*Mmc*-95010 [23] and pMV158, a small, broad-host-range plasmid, originally isolated from *Streptococcus agalactiae* that is considered the prototype of the rolling circle replicating plasmid family [45] (Figure 3A). It consists of two CDSs transcribed in the same direction, followed by an inverted repeat sequence ended by a stretch of thymidine residues that is typical of rho-independent transcription terminators (Tcr; Figure 3A).

**Figure 4:**
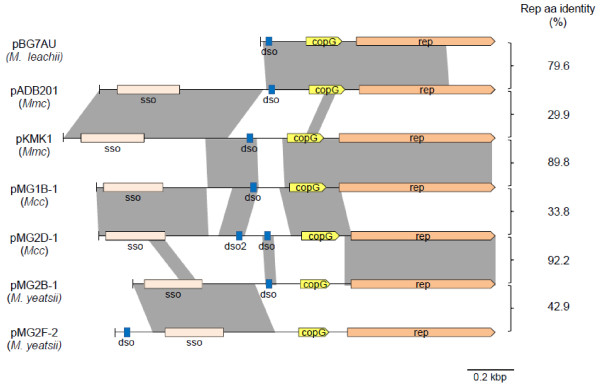
**Pairwise comparisons of nucleotide sequences of mycoplasma plasmids.** Aligned regions with significant levels of similarity are shaded in grey. Relevant loci are indicated. sso, putative single strand origin; dso, double strand origin. Comparisons were generated with the Artemis Comparison Tool, ACT [39]. Percentages of identical amino acids between pairs of Rep are indicated on the right.

The first CDS encodes a 43–53 aa polypeptide predicted to be the transcriptional regulator CopG by homology to that of pMV158 (Figure 3C). Despite the low similarity level between the predicted polypeptides, the key amino-acids within a predicted helix-turn-helix structure are conserved (Figure 3C). In pMV158, the CopG protein regulates the plasmid copy number through the control of *coprep* mRNA synthesis. Furthermore, the copy number of pMV158 is also controlled through a small counter-transcribed RNA (ctRNA) [47]. In agreement with this type of regulation, the corresponding transcription signals (promoter Pct and rho independent terminator Tct; Figure 4A) were predicted on the complementary strand in between the two CDS of the various plasmids (Additional file 4: Figure S1).

The second CDS encodes a 196–225 aa polypeptide that was annotated as the replication protein, Rep in pADB201, again by homology to pMV158. All predicted Rep proteins shared a Rep2 domain (Plasmid replication protein, pfam01719). These Rep proteins are known to function as topoisomerases that nick the positive strand at the leading strand origin of replication (*dso*) during rolling-circle replication [48]. Multiple sequence alignments revealed that the Rep proteins of mycoplasma plasmids shared five conserved motifs (I to V) initially described in the Rep proteins from the pMV158 family [46] (Figure 4D). Consistent with this finding, a double-strand origin (*dso*) typical of pMV158 family was identified upstream of *copG* (Figure 4B). These *dso* shared a conserved cleavage site TACTAC(C)G/A between two inverted repeats. The other replication origin, the lagging-strand initiation site (*sso*), was also predicted upstream of the *dso* by analogy with results obtained for other mycoplasma plasmids [22,23] (data not shown). Therefore, replication of all mycoplasma plasmids is likely to be driven through a rolling-circle mechanism by a Rep protein of the pMV158 family type.

### Mosaic structure of the mycoplasma plasmids is indicative of recombination events

**Figure 5:**
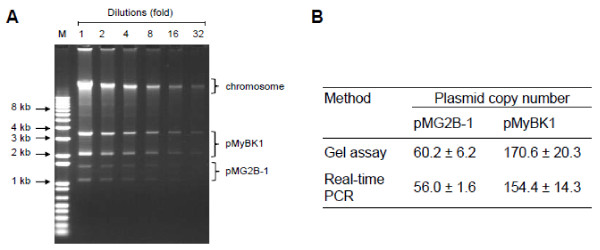
**Analysis of plasmid content of *Mycoplasma yeatsii *type strain GIH (TS).A.** Agarose gel electrophoresis of total DNA. Lanes were loaded after twofold dilution series of the DNA preparation obtained as described in Methods. Bands corresponding to the chromosome and the 2 plasmids are identified. Lane M, DNA ladder. **B.** Estimated plasmid copy number of pMyBK1 and pMG2B-1 as estimated by gel assay (Panel A) and relative real-time PCR as described in Methods.

In spite of a conserved structure, multiple pair-wise DNA sequence comparisons indicated that mycoplasma plasmids are in fact a mosaic of *rep, dso, copG*, and *sso* blocks. This was evidenced by the occurrence of several local regions of homology detected by using the BLAST program (Figure 5). Pairs of plasmids that show a high level of identity for the Rep sequence (e.g. pKMK1 and pMG1B-1; pMG2D-1 and pMG2B-1) do not necessarily share a high degree of identity for the region upstream of *copG*. Interestingly, high sequence identity for the region spanning *sso* was found to be indicative of plasmids being hosted by the same mycoplasma species. For instance, the following plasmid-pairs, pADB201 and pKMK1, pMG1B-1 and pMG2D-1, and pMG2B-1 and pMG2F-2 were isolated from *Mmc, Mcc,* and *M. yeatsii*, respectively (Figure 5). This result is consistent with the fact that during replication this region interacts with chromosome-encoded components [18]. Further degrees of mosaicism were found in particular cases such as for pMG2D-1, in which two putative *dso* showing sequence heterogeneity are found. Other examples of genetic variability are the small size of pBG7AU and the unusual location of the *dso* in pMG2F-2. Such a mosaic structure is clearly indicative of successive recombination events between replicons.

### pMyBK1 is a unique representative of a new replicon family

As indicated above, *M. yeatsii* strain GIH TS was the only strain that yielded a banding pattern of extrachromosomal DNA that suggested the presence of two distinct plasmids (Figure 5A). The small plasmid, pMG2B-1, was shown to belong to the pMV158 family like all other mycoplasma plasmids (Figure 3). In contrast, the larger plasmid (3,432 bp) named pMyBK1 (GenBank Accession number EU429323; [25]) has a genetic organization that sets it apart from the other mycoplasma plasmids. Initial database searches using pMyBK1 sequence as a query indicated low identity with other plasmids and prompted us to further analyze this plasmid that might represent a new family of replicons.

First, the relative copy number of each plasmid of *M. yeatsii* GIH^T^, pMG2B-1 and pMyBK1, was evaluated by two different methods (gel assay and real-time PCR). Both methods yielded similar results with estimated copy number of 154–170 copies/cell and of 56–60 copies/cell for pMyBK1 and pMG2B-1, respectively (Figure 5B). Such a difference strongly suggests that the two plasmids have distinct replication and /or regulation systems. Together the 2 *M. yeatsii* plasmids represent a total extrachromosomal DNA amount of 636 kbp per cell, which is approximately 37% of the total cell DNA.

Next, the genetic structure of pMyBK1 was analyzed. The 2 CDSs found in the pMyBK1 sequence (CDSA and B, encoding polypeptides of respectively 519 and 272 aa) showed no homolog with other mycoplasma plasmids (Figure 2A). The presence of a 192-bp intergenic region between the CDSs as well as the predicted rho-independent transcription terminator immediately downstream of each CDS strongly suggests that the 2 CDSs are transcribed independently rather than as a single operon. The deduced amino acid sequence of pMyBK1 CDSA exhibits low but significant similarity with mobilization proteins of various bacteria. The N-terminal part of the CDSA protein contains a Mob/Pre domain (pfam01076) typical for relaxases of the MobV superfamily that includes proteins involved in conjugative mobilization and plasmid intramolecular recombination [49]. Sequence alignments with representatives of the MobV family clearly showed that the CDSA protein did possess the three conserved motifs of the family [50] (data not shown). Subsequent phylogenetic analyses of the CDSA polypeptide with the complete set of MobV proteins described by Garcillan-Barcia [51] classified the pMyBK1 protein within the MobV4 relaxase family (data not shown).

In contrast to CDSA, no functional domain or characteristic secondary structure was identified in the CDSB-encoded protein. Blast searches revealed that the CDSB protein of pMyBK1 shared significant homology with five chromosome-encoded proteins of *Mcc*, strain California Kid, or *M. leachii*, strain PG50 and 99/014/6 but with no known associated function.

### Identification of the replication protein and the mode of replication of pMyBK1

Since none of the pMyBK1-encoded proteins share homology to known replication proteins, CDSA and CDSB were both regarded as putative candidates. To identify the replication protein and delineate the replication region of pMyBK1, a series of deletion and frameshift mutations were introduced in a shuttle plasmid (*E. coli*/*M. yeatsii*), named pCM-H, that was constructed by combining pMyBK1 to a colE1 replicon carrying the *tetM* tetracycline resistance gene as the selection marker (Figure 2A). The mutated plasmids were then introduced into a plasmid-free *M. yeatsii* strain (#13156 from the Anses collection) by PEG-transformation, and their replication capacity was measured by the number of resulting tetracycline resistant colonies. Plasmids pCM-P and pCM-K1 contain respectively CDSA and CDSB, associated with the flanking intergenic regions (Figure 2A). No transformant was obtained with pCM-P, confirming that CDSA, which encodes a putative Mob protein (see before), is not the replication protein and that none of the intergenic regions is sufficient to sustain plasmid replication. In contrast, the replication of pCM-K1 in *M. yeatsii* was abolished after introducing a frameshift mutation that disrupts CDSB (pCM-K1 ΔB in Figure 2A). This strongly argues for CDSB encoding the replication protein of pMyBK1, a result that confirms recent findings [25]. Successive reductions of the region downstream of CDSB, including the GC rich sequence located immediately upstream of CDSA of the native plasmid, led to a minimal replicon pCM-K4 of 1,297 bp (Figure 2A). In pCM-K4, the region downstream of CDSB is characterized by the presence of two sets of direct repeats. In addition, a 44-bp partially palindromic sequence with the potential to form a stable stem-loop structure (ΔG = −8.71 kcal/mol) is located immediately downstream of the direct repeat region. Interestingly, this structure was found to be essential for plasmid replication as deletion of the stem-loop 5’arm in pCM-K5 totally abolished plasmid replication (Figure 3A).

Detection of single-stranded (ssDNA) intermediates, generated during replication, is the hallmark of plasmids replicating via a rolling-circle mechanism [40,52]. After treatment of some of the DNA samples with ssDNA-specific nuclease S1, total DNAs from M. *yeatsii* GIH TS were separated by agarose gel electrophoresis before being transferred to nylon membranes under non-denaturating conditions. Hybridization with the pMyBK1 probe could only be detected when S1-nuclease treatment was omitted (Additional file 5: Figure S2). The hybridization signal was completely absent in the corresponding, S1-nuclease treated samples (Additional file 5: Figure S2). These results confirmed the existence of ssDNA intermediates and indicate that pMyBK1 probably replicates via the RCR mechanism. Since CDSB protein has no similarity with any known replication protein, pMyBK1 is therefore considered as the first member of a new RCR replicon family.

### Host specificity of pMyBK1

The lack of significant similarity between the putative Rep of pMyBK1 and the Rep proteins from other mycoplasma plasmids confirms that pMyBK1 belongs to a previously unknown class of RCR plasmids. However, the fact that pMyBK1 is hosted by a mycoplasma species (*M. yeatsii*) sharing a common host (goat) and body site (ear canal) with other ruminant mycoplasmas [53,54] raises the question of the putative dissemination of this plasmid. Therefore, the ability of pMyBK1 derivatives to replicate in various mollicute species of the *Mycoplasma* and *Spiroplasma* genera was evaluated. Using the standard PEG-transformation protocol, the pMyBK1-derivatives pCM-K3/4 (Figure 2B) were successfully introduced into the following plasmid-free strains: *M. yeatsii* #13156, *M. putrefaciens* KS1 TS, *M. leachii* PG50 TS and *Mcc* California kid TS. The autonomous replication of the pMyBK1 derivatives in these species was confirmed by plasmid purification and back-transformation of *E. coli* with the purified plasmids. Transformation of *Mmc* with pCM-K3/4 also yielded many tetracycline resistant transformants, but no free plasmid could be detected despite the positive PCR amplification of CDSB. These results suggest an integration of the pMyBK1 derivative into the host chromosome of this species, as it has been previously described for *oriC* plasmids [55]. Attempts to transform *M. mycoides* subsp. *mycoides* or *Spiroplasma citri* with pCM-K3 repeatedly failed. Interestingly, we also showed that pMyBK1 not only replicated in various mycoplasma species but was also able to express heterologous genes. The spiralin gene encoding the major surface protein of *S. citri* was inserted into the *Eco*RI site of pCM-K3 and the resulting plasmid pCM-K3-spi (Figure 2A) was successfully introduced into *M. yeatsii* GIH TS and *Mcc* California Kid. Expression of spiralin by the transformants was demonstrated by immunoblotting (Additional file 6: Figure S3 for *Mcc* transformants, data not shown for *M. yeatsii* transformants). These results confirm and extend recently published results [25] indicating that pMyBK1 derivatives can be used as expression vectors in mycoplasma species of veterinary importance.

### General phylogeny of Rep sequences from mycoplasma plasmids

**Figure 6:**
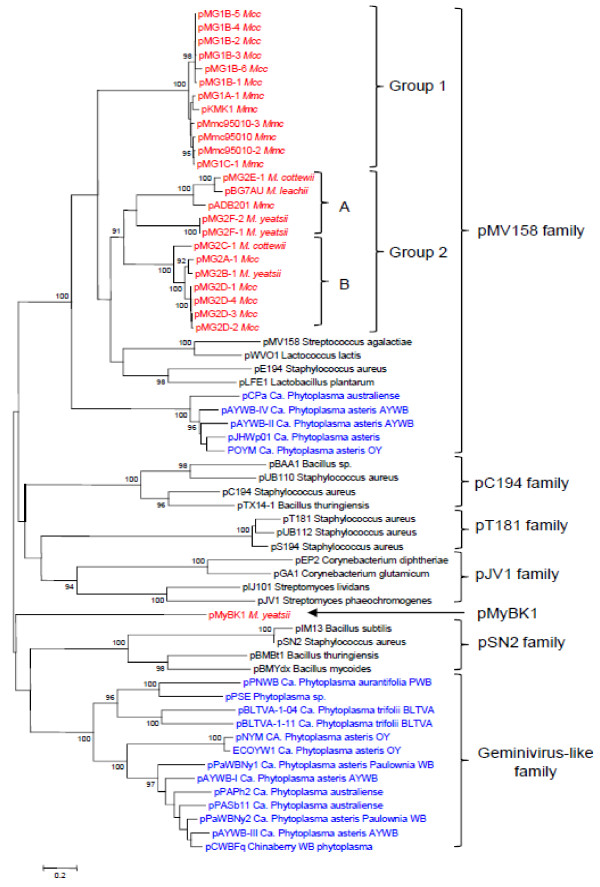
**Phylogenetic relationships among RCR plasmids based on Rep protein sequences.** The evolutionary history was inferred using the Neighbor-Joining method [56]. The percentage of replicate trees in which the associated sequences clustered together in the bootstrap test (1000 replicates) are shown next to the branches [57]. Plasmids from mollicutes are indicated in red (mycoplasmas) and blue (phytoplasmas).

Based on the availability of 25 Rep sequences of mycoplasma plasmids (Additional file 3: Table S3), it was possible to address how these sequences cluster in the phylogenetic tree constructed with a set of sequences including representatives of RCR plasmids from both *Mollicutes* and *Firmicutes* (Figure 6). A set of 62 amino acids sequence corresponding to the replication protein of 25 mycoplasma plasmids and of 37 representatives of the major RCR plasmid families, including those of the phytoplasma plasmids was selected for constructing the phylogenetic tree. Phylogenetic analyses confirmed that, except for pMyBK1, all mycoplasma plasmids could be grouped within the pMV158 family (Figure 6). This result is consistent with the prediction, in these Rep sequences, of a Rep2 domain typical of this plasmid family. Yet, mycoplasma plasmids do not form a single, coherent group in this family but instead cluster into two distinct branches designated as groups 1 and 2. Rep proteins from groups 1 and 2 share only limited similarities and, the most divergent members in these groups are more distant between each other than they are from the streptococcal pMV158. Group 1 consists of highly similar proteins (identity ranging from 88 to 100%) and includes Rep proteins from *Mmc* and *Mcc* plasmids. Conversely, group 2 is more heterogeneous and includes Rep proteins from *M. leachii, M. yeatsii, M. cottewii, Mmc* and *Mcc* plasmids. Further phylogenetic analyses showed that group 2 could be split into two statistically-supported subgroups (2A and 2B).

It is noteworthy that a large group of phytoplasma plasmids also clusters within the pMV158 family. Nevertheless, the Rep proteins of phytoplasma plasmids are more closely related to Rep of mobile elements from non-mollicute bacteria than to those of mycoplasma plasmids. In addition, the Rep of phytoplasma plasmids are characterized by a C-terminal part having a helicase domain, which is absent in the Rep of mycoplasma plasmids.

## Conclusions

This study was performed in the context of (i) conflicting reports regarding the prevalence of plasmids in mycoplasma species [3,24] and of (ii) the quest for MGE that may have served as genetic vehicles resulting in the high level of HGT reported among ruminant mycoplasmas [58]. We found a rather high prevalence of plasmids in species belonging to the *M. mycoides* cluster and, in contrast, a lack of plasmids in the *M. bovis-M. agalactiae* group. Therefore, these plasmids are unlikely to contribute by themselves to a significant part of the reported HGT, and therefore the role of other MGE, including ICEs, remains to be evaluated.

The present study has considerably increased our knowledge about the genetic organization of mycoplasma plasmids adding 21 new sequences to a repertoire of only 5 in the databases. With the exception of the previously reported pMyBK1 replicon, all the mycoplasma plasmids belong to the pMV158 family. As these plasmids only encode two genes, one essential for replication initiation and the other for control of copy number, they do not carry any accessory gene that may confer a new phenotype to the recipient cell.

The alignment of *rep* plasmid sequences resulted in a tree that does not fit the 16S rDNA phylogeny of the host species. For instance, the Rep proteins of *Mcc* pMG1B-1 and pMG2A-1 fall into two distinct groups whereas those of *Mcc* pMG2A-1 and *M. yeatsii* pMG2B-1 are almost identical (Figure 6, Table S3). Incongruence between plasmid and chromosomal gene phylogenies has often been reported in bacteria and interpreted as the result of lateral plasmid transfer between diverse species [59,60]. In addition, plasmid phylogeny has probably been blurred by recombination events that resulted in a mosaic structure (Figure 4). The occurrence of several mycoplasma species within the same host (i.e. small ruminants) might have facilitated horizontal plasmid transfer within this bacterial genus. The driving force for this extrachromosomal inheritance has yet to be further studied taking into account the apparent lack of beneficial traits by the recipient species. Mechanisms underlying the transfer of plasmids between strains of ruminant mycoplasmas have yet to be deciphered since, the mycoplasma pMV158-like plasmids, like a majority of plasmids should be considered as non mobilizable/non self-transmissible according to the classification of Smilie et al. [61]. Their small size favors transfer mechanisms like transduction, natural transformation and co-integration in mobile elements.

The topology of the *rep* phylogenetic tree (Figure 6) is not consistent with the idea of a common plasmid ancestor that would have been vertically inherited in both phytoplasma and mycoplasma clades. Moreover, the clear-cut clustering of mycoplasma plasmids into separate branches supports the hypothesis of several, rather than a single, mycoplasma plasmid ancestors. Using the clustering of *rep* sequences, we propose a new nomenclature system that applies to all currently described mycoplasma and phytoplasma plasmids. This classification does not take into account the plasmid host as these elements are transmissible from one species to another. As the spiroplasma plasmids do not carry a *rep* sequence showing a significant homology with those described here (Figure 6), they cannot be included in this nomenclature.

While this paper was under review, Kent et al. published a study showing the use of pMyBK1 as a shuttle vector for heterologous gene expression in *M. yeatsii*[25]*.* We confirm that pMyBK1 represents a novel RCR plasmid family and that its derivatives can be used as gene vectors to express cloned genes not only in *M. yeatsii*[25] but also in three other ruminant mycoplasmas. This result is not trivial in a group of organisms for which the genetic toolbox is very limited. The pMyBK1 plasmid has a small size, lacks any CDS homologous to genes for mating pair formation but encodes a relaxase belonging to the MobV class. These features argue for a mobilizable rather than conjugative nature of the plasmid [25,62]. The fact that pMyBK1 was only detected in *M. yeatsii* is inconsistent with the finding that it replicates in mycoplasma species other than *M. yeatsii*, at least when introduced experimentally. Two hypotheses would explain this apparent contradiction. One is that the transfer of pMyBK1 is a rare event and hence, the number of strains screened was not large enough to detect additional pMyBK1-related plasmids. The other is that pMyBK1 would not be transferred in vivo or would not be stably maintained once transferred.

## Abbreviations

(HGT): Horizontal gene transfer; (MGE): Mobile genetic elements; (ICEs): Integrative and conjugative elements; (*Mmc*): *M*. *mycoides* subsp. *capri*; (*Mcc*): *M. capricolum* subsp. *capricolum*; (CDS): Coding Sequence; (dso): Leading strand origin of replication; (sso): Lagging-strand initiation site; (RCR): Rolling-circle replication; (PEG): Polyethylene glycol.

## Competing interests

All the authors declare no competing interests.

## Authors’ contributions

MB, FT, VM and ED-F carried out most of the experiments. MB, FT, CC, ED-F and AB participated in the design of the study. MB and FT drafted the manuscript, all authors made suggestions for improvement. All authors participated in the data analysis. FT, CC and AB coordinated the study. All authors read and approved the final manuscript.

## Supplementary Material

Additional file 1 **Table S1.** Additional file 5. Click here for file

Additional file 2 **Table S2.** History of mycoplasma strains and plasmid screening. Click here for file

Additional file 3 **Table S3.** Pairwise nucleic sequence identities between mycoplasma plasmids. Global alignments of the full-length nucleic sequence of mycoplasma plasmids were accomplished using a Needleman–Wunsch algorithm implemented in the Needleall program (Needleman & Wunsch, 1970). Identity percents are indicated. Rep group refers to Rep phylogeny (see Figure 6).** Table S3.** Pairwise nucleic sequence identities between mycoplasma plasmids. Global alignments of the full-length nucleic sequence of mycoplasma plasmids were accomplished using a Needleman–Wunsch algorithm implemented in the Needleall program (Needleman & Wunsch, J Mol Biol 1970;48:443-53). Identity percents are indicated. Rep group refers to Rep phylogeny (see Figure 6). Click here for file

Additional file 4 **Figure S1.** Nucleotide sequences of the predicted ctRNA coding strands. The counter-transcripts were first identified by analogy with those of pMV158 or its derivative pLS1. These ctRNA overlap the *rep* gene start and have a length of only a few tens of nucleotides. Using the consensus sequence TTGACA – (N17) –TG-N-TATAAT for the promoter, putative promoters were identified in the aligned sequences. Putative *Pct* promoters are indicated with the -35 and -10 regions in bold and underlined letters. Arrows indicate inverted repeats of the putative *rho* independent terminators. The ctRNA of pLS1 (*rnaII*) is shown as proposed by del Solar et al. [46] with an arrowhead indicating the possible transcriptional initiation site. The box CAT indicates the initiation codon of the *rep* gene that is encoded on the complementary DNA strand. Click here for file

Additional file 5 **Figure S2.** Detection of pMyBK1 ssDNA intermediates by Southern blot hybridization. Total DNA from *Mycoplasma yeatsii* type strain GIH TS (lane 1-2) was analyzed on a 0.8% agarose gel (A) with (+) or without (-) prior S1 nuclease treatment. Southern blot (B) was performed with digoxigenin-labeled pMyBK1 probe under non-denaturing conditions. M, DNA ladder. Click here for file

Additional file 6 **Figure S3.** Expression of spiralin in Mcc using pMyBK1 derivatives. Whole cell dot immunoblot of 12 Mcc transformants harboring the spiralin expression vector pCM-K3-spi (a) or the empty vector pCM-K3 (b). Mycoplasma cells were applied to a nitrocellulose membrane and probed with rabbit anti-spiralin antibodies and anti-rabbit IgG peroxidase conjugate. Click here for file
