# Supplementary material for: Distribution and diversity of mycoplasma plasmids: lessons from cryptic genetic elements
Source: BMC Microbiol. 2012 Nov 12;12:257. doi: 10.1186/1471-2180-12-257 (PMC3541243; doi:10.1186/1471-2180-12-257)
Supplement: Additional file 1 — Table S1. Additional file 5. [file 1471-2180-12-257-S1.docx]

**Table S1:** List of oligonucleotides used in this study.

| Target / purpose | Name | Sequence (5’-3’) | Temp. ^(c)^ | Size |
| --- | --- | --- | --- | --- |
| Rep / plasmid detection in *Mcc* strains | Mcc_Rep_F | TTGTCCTTTATATTATACCATAAT^(a)^ | 53°C | 709 bp |
|  | Mcc_Rep_R | TGCTATTTACTGACTCTATTTTG^(a)^ |  |  |
| Rep / plasmid detection in *Mmc* strains | Mmc_Rep_F | AGTTTCTAAGATGATGAATTTTTAA ^(a)^ | 51°C | 1,681 bp^(b)^ |
|  | Mmc_Rep_R | TCCATTACATCCTTTTTCCTTT^(a)^ |  |  |
| *glpK* (*M.yeatsii*) / SybrGreen quantification | glpkF | CGTGGAGCTATTTTCGGATT | 62°C | 123 bp |
|  | glpkR | CCATTGCTTCAACAACGTCA |  |  |
| pMG2B1 *rep* */* SybrGreen quantification | pMG2B1-F | GACGTCAATCCAGACGGAAC | 62°C | 90 bp |
|  | pMG2B1-R | CGACGCTTTCTATTCCTTGC |  |  |
| pMyBK1 *cdsB /* SybrGreen quantification | cdsB-F | CAGCAAAACAAAGAGAACAAGC | 62°C | 87 bp |
|  | cdsB-R | TGGTCAATGAAACTTGAAGCA |  |  |
| pMyBK1/construction of pCM-H vector^(d)^ | pMyBK1-1F1 | AAAAAA**AGATCT**AGAAAATAGCTTAAATATCAGCTTTAATGC | 56°C | 3,456 bp |
|  | pMyBK1-1R1 | AAAAAA**AGATCT**AAATTAATAACTTATAAAGGTCATTTCAAG |  |  |
| pMyBK1/ construction of pCM-P vector^(d)^ | pMyBK1-1F1 | See above | 56°C | 2,610 bp |
|  | pMyBK1-1R2 | AAAAAA**AGATCT**TAAGTTGTAGATTCATTATATAAGCCTTCT |  |  |
| pMyBK1/ construction of pCM-C vector^(d)^ | pMyBK1-2F1 | AAAAAA**AGATCT**AAAATACCATTGACTGGTATTTTATTTTTG | 56°C | 3,456bp |
|  | pMyBK1-2R1 | AAAAAA**AGATCT**TACATTTTTAGGGTATTTTTGTATCCTGTC |  |  |
| pMyBK1/ construction of pCM-K1 vector^(d)^ | pMyBK1-2F1 | See above | 56°C | 1,862 bp |
|  | pMyBK1-2R2 | AAAAAA**AGATCT**GTACTTGATAACTCATTATTACTACCTACT |  |  |
| pMyBK1/ construction of pCM-K2 vector^(d)^ | pMyBK1-2F2 | AAAAAA**AGATCT**AGAAGGCTTATATAATGAATCTACAACTTA | 56°C | 1,686 bp |
|  | pMyBK1-2R2 | See above |  |  |
| pMyBK1/ construction of pCM-K3 vector^(d)^ | pMyBK1-2F1 | See above | 56°C | 1,635 bp |
|  | pMyBK1-2R3 | AAAAAA**AGATCT**TGTGAAATTGTTAGAAGCGTAGTGGATGA |  |  |
| pMyBK1/ construction of pCM-K4 vector^(d)^ | pMyBK1-2F1 | See above | 56°C | 1,251 bp |
|  | pMyBK1-2R4 | AAAAAA**AGATCT**CAAAATAAACTAAAAAGAAAACAAAAAGTGGTTGC |  |  |
| pMyBK1/ construction of pCM-K5 vector^(d)^ | pMyBK1-2F1 | See above | 56°C | 1,219 bp |
|  | pMyBK1-2R5 | AAAAAA**AGATCT**AGTGCTTTAGCAGTAGCAAAAGCTTTTTGCT |  |  |
| pCM-K1/disruption of cdsB in pCM-ΔK1^(d)^ | DeltacdsB-F | AAAAAA**CTCGAG**TTCATTACAAAAACCAACTAGAAATGGTAC | 60°C | 7,404 bp |
|  | DeltacdsB-R | AAAAAA**CTCGAG**CTCTATCTATTGTTATTCCTGAAG |  |  |
| Spiralin gene / Gene expression in vectors^(d)^ | SpiERI-F | AAATAA**GAATTC**AAAGTTAGTGAACAAGAAAACAGTG | 55°C | 999 bp |
|  | SpiERI-R | AAAAGT**GAATTC**TTATCCTGCATTTGCTGGTGC |  |  |

^(a)^ The complementary reverse oligonucleotides were used to amplify back-to-back the remaining part of the circular plasmid.

^(b)^ Size of the amplicon as estimated on p*Mmc*95010.

^(c)^ Hybridization temperature used for PCR assays.

^(d)^ The BglII, XbaI and EcoRI sites in the primer sequences that were used for cloning are in bold
