# Supplementary material for: Distribution and diversity of mycoplasma plasmids: lessons from cryptic genetic elements
Source: BMC Microbiol. 2012 Nov 12;12:257. doi: 10.1186/1471-2180-12-257 (PMC3541243; doi:10.1186/1471-2180-12-257)
Supplement: Additional file 4 — Figure S1. Nucleotide sequences of the predicted ctRNA coding strands. The counter-transcripts were first identified by analogy with those of pMV158 or its derivative pLS1. These ctRNA overlap the rep gene start and have a length of only a few tens of nucleotides. Using the consensus sequence TTGACA – (N17) –TG-N-TATAAT for the promoter, putative promoters were identified in the aligned sequences. Putative Pct promoters are indicated with the -35 and -10 regions in bold and underlined letters. Arrows indicate inverted repeats of the putative rho independent terminators. The ctRNA of pLS1 (rnaII) is shown as proposed by del Solar et al. [46] with an arrowhead indicating the possible transcriptional initiation site. The box CAT indicates the initiation codon of the rep gene that is encoded on the complementary DNA strand. [file 1471-2180-12-257-S4.docx]

pMy_GIH_2 (rev 963-860)

**TTGTTT**TTTTATTCATATTT**TG**A**TAAAAT**TAAATGGATAAAAATTCTCACTAAAGGTTTTATCATCAGCACTAAAAAGGAGTAGGTTTACCTGCTCTTTTTTT

pKMK1 (rev 308-208)

**TTACAG**TTTGTTTCATATTC**TG**A**TACAAT**ATAAGGGACAAGAAAATTCAAAATTCATCATTTTAAAAAACTTAGAAACTCTTATTCGGGTTTCTTTTTTTT

pMmc-95010 (1379-1483)

**TTACAA**TTTGTTTCATATTC**TG**A**TACAAT**ATAAAGGACAAGAAAATTTAAAAAATTAAAAATTCATCATCTTAGAAACTCTTATTCGGGTTTCTTTTTTTTGTTT

pADB201 (rev 470-378)

**TTATAT**TAGTTGACATTATC**TG**C**TATAAT**ATAAGGGATAAAAAATATTTGTTTTACAAAGAAGCTTATTAGAGAACTAAAGCTTCTTTTTTTT

pBG7AU (rev 468-378)

**TAATAT**TAGTTGACATTATT**TG**C**TATAAT**ATAAGGGATAAAAAATATTTGTTTTACAAAGAAGCTTATTAGAGAACTAAAGCTTCTTTTTTTT

pMV158 (rev 871-785)

**TTGCTT**TTTCTTTAGCCATAAAG**TATAAT**ATACCCAAGAAATGTTTGACTTTAGCCAGTGGCTGCCAGCACGGCTTTTTTTATTTTT

**Figure S1. Nucleotide sequences of the predicted ctRNA coding strands**. : the counter-transcripts were first identified by analogy with those of pMV158 or its derivative pLS1. These ctRNA overlap the *rep* gene start and have a length of only a few tens of nucleotides. Using the consensus sequence TTGACA – (N17) –TG-N-TATAAT for the promoter, putative promoters were identified in the aligned sequences. Putative *Pct* promoters are indicated with the -35 and -10 regions in bold and underlined letters. Arrows indicate inverted repeats of the putative *rho* independent terminators. The ctRNA of pLS1 (*rnaII*) is shown as proposed by del Solar et al [47] with an arrowhead indicating the possible transcriptional initiation site. The box CAT indicates the initiation codon of the *rep* gene that is encoded on the complementary DNA strand.
