# Supplementary material for: Distribution and diversity of mycoplasma plasmids: lessons from cryptic genetic elements
Source: BMC Microbiol. 2012 Nov 12;12:257. doi: 10.1186/1471-2180-12-257 (PMC3541243; doi:10.1186/1471-2180-12-257)
Supplement: Additional file 5 — Figure S2. Detection of pMyBK1 ssDNA intermediates by Southern blot hybridization. Total DNA from Mycoplasma yeatsii type strain GIH TS (lane 1-2) was analyzed on a 0.8% agarose gel (A) with (+) or without (-) prior S1 nuclease treatment. Southern blot (B) was performed with digoxigenin-labeled pMyBK1 probe under non-denaturing conditions. M, DNA ladder. [file 1471-2180-12-257-S5.pptx]

## Slide 1
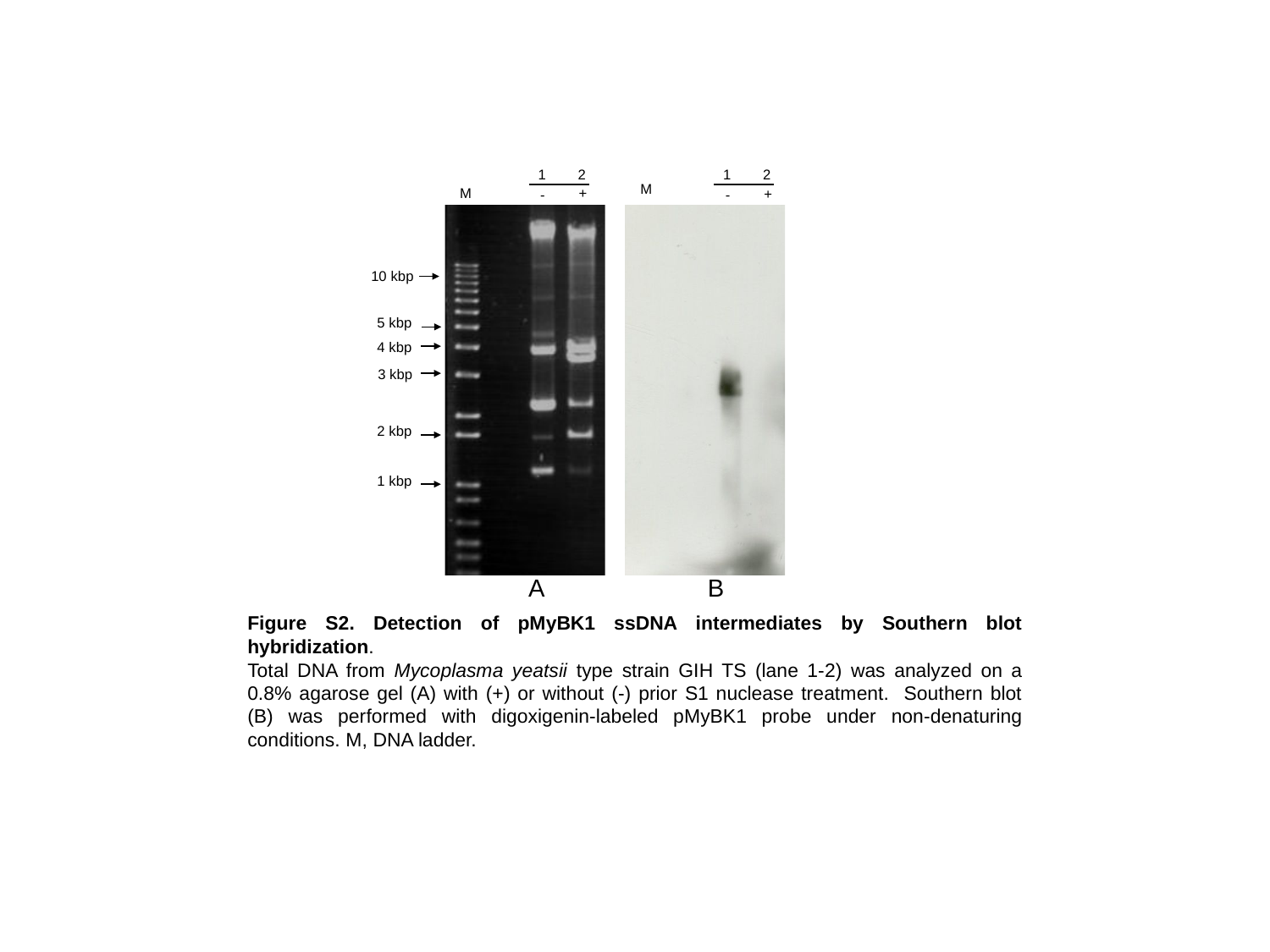

1
2
+
-
1
2
+
-
M
M
10 kbp
5 kbp
4 kbp
3 kbp
2 kbp
1 kbp
A
B
Figure S2. Detection of pMyBK1 ssDNA intermediates by Southern blot hybridization.
Total DNA from Mycoplasma yeatsii type strain GIH TS (lane 1-2) was analyzed on a 0.8% agarose gel (A) with (+) or without (-) prior S1 nuclease treatment. Southern blot (B) was performed with digoxigenin-labeled pMyBK1 probe under non-denaturing conditions. M, DNA ladder.
