# Supplementary material for: Distribution and diversity of mycoplasma plasmids: lessons from cryptic genetic elements
Source: BMC Microbiol. 2012 Nov 12;12:257. doi: 10.1186/1471-2180-12-257 (PMC3541243; doi:10.1186/1471-2180-12-257)
Supplement: Additional file 6 — Figure S3. Expression of spiralin in Mcc using pMyBK1 derivatives. Whole cell dot immunoblot of 12 Mcc transformants harboring the spiralin expression vector pCM-K3-spi (a) or the empty vector pCM-K3 (b). Mycoplasma cells were applied to a nitrocellulose membrane and probed with rabbit anti-spiralin antibodies and anti-rabbit IgG peroxidase conjugate. [file 1471-2180-12-257-S6.pptx]

## Slide 1
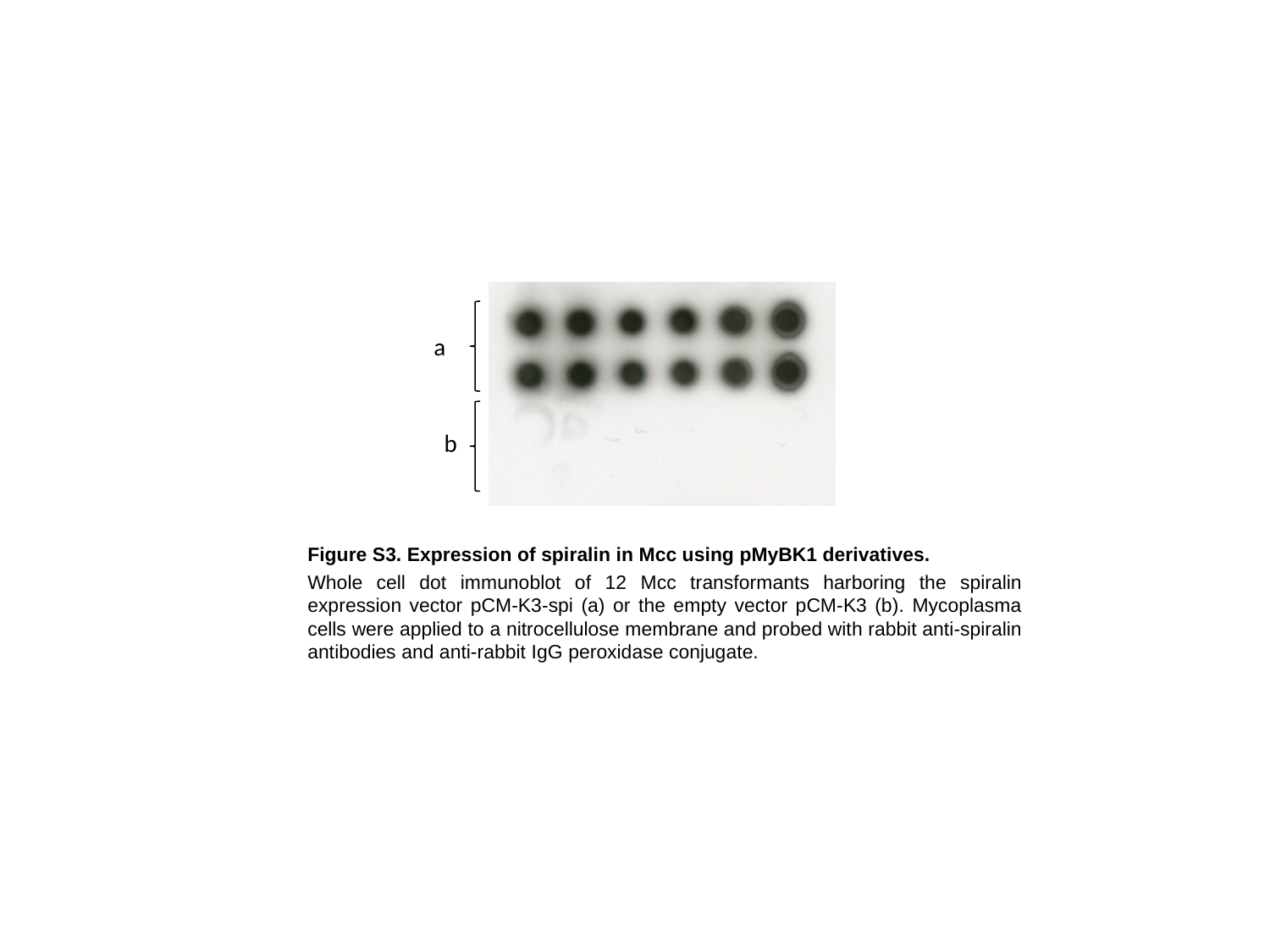

a
b
Figure S3. Expression of spiralin in Mcc using pMyBK1 derivatives.
Whole cell dot immunoblot of 12 Mcc transformants harboring the spiralin expression vector pCM-K3-spi (a) or the empty vector pCM-K3 (b). Mycoplasma cells were applied to a nitrocellulose membrane and probed with rabbit anti-spiralin antibodies and anti-rabbit IgG peroxidase conjugate.
